# Serum Metabolite Biomarkers for Predicting Residual Feed Intake (RFI) of Young Angus Bulls

**DOI:** 10.3390/metabo10120491

**Published:** 2020-11-30

**Authors:** Aidin Foroutan, Carolyn Fitzsimmons, Rupasri Mandal, Mark V. Berjanskii, David S. Wishart

**Affiliations:** 1 Department of Agricultural Food and Nutritional Science, University of Alberta, Edmonton, AB T6G 2P5, Canada; aidin@ualberta.ca (A.F.); cfitzsim@ualberta.ca (C.F.); 2 Department of Biological Sciences, University of Alberta, Edmonton, AB T6G 2E9, Canada; rmandal@ualberta.ca (R.M.); mb1@ualberta.ca (M.V.B.); 3 Agriculture and Agri-Food Canada, Edmonton, AB T6G 2P5, Canada; 4 Department of Computing Science, University of Alberta, Edmonton, AB T6G 2E8, Canada

**Keywords:** residual feed intake, serum, metabolite, biomarker, Angus bulls

## Abstract

Residual feed intake (RFI) is a feed efficiency measure commonly used in the livestock industry to identify animals that efficiently/inefficiently convert feed into meat or body mass. Selection for low-residual feed intake (LRFI), or feed efficient animals, is gaining popularity among beef producers due to the fact that LRFI cattle eat less and produce less methane per unit weight gain. RFI is a difficult and time-consuming measure to perform, and therefore a simple blood test that could distinguish high-RFI (HRFI) from LRFI animals (early on) would potentially benefit beef farmers in terms of optimizing production or selecting which animals to cull or breed. Using three different metabolomics platforms (nuclear magnetic resonance (NMR) spectrometry, liquid chromatography-tandem mass spectrometry (LC-MS/MS), and inductively coupled plasma mass spectrometry (ICP-MS)) we successfully identified serum biomarkers for RFI that could potentially be translated to an RFI blood test. One set of predictive RFI biomarkers included formate and leucine (best for NMR), and another set included C4 (butyrylcarnitine) and LysoPC(28:0) (best for LC-MS/MS). These serum biomarkers have high sensitivity and specificity (AUROC > 0.85), for distinguishing HRFI from LRFI animals. These results suggest that serum metabolites could be used to inexpensively predict and categorize bovine RFI values. Further validation using a larger, more diverse cohort of cattle is required to confirm these findings.

## 1. Introduction

Residual feed intake (RFI) is a livestock feed efficiency measure defined as the difference between an animal’s actual feed intake and its expected feed requirements for maintenance and growth over a specific time period. RFI is independent of growth characteristics such as body weight (BW) and average daily gain (ADG) [1,2]. RFI measurements are laborious, expensive, and time-consuming as they require measuring an individual animal’s BW and feed intake over a period of 76 days [1,3]. The RFI value is typically calculated over a group or herd of cattle, where the mean RFI for that group is defined as 0 kg/day. Low-RFI (LRFI) animals eat less than average, while high-RFI (HRFI) animals eat more than average. For example, an animal with an RFI value of −1.9 kg/day eats 1.9 kg/day less than the mean of 0 kg/day and is considered to be a LRFI or a feed efficient animal. Selection for LRFI animals is gaining popularity among beef producers because LRFI cattle eat less per unit weight gain. Another positive attribute of LRFI cattle is that they produce less methane. Methane is a greenhouse gas, which is produced by ruminants during digestion and fermentation [4]. Livestock are responsible for the emission of ~18% of the global anthropogenic output of greenhouse gases [4,5], and therefore reducing their carbon footprint is a key factor in reducing global warming. Several studies have shown that selecting for LRFI cattle is associated with reduced methane production [6,7]. Indeed, compared with HRFI cattle, 25–28% lower methane production in LRFI animals has been reported [6,7]. Therefore, selection for feed efficiency can favor both the farmer (decreased production costs) and the environment (lower methane and manure production). In addition, RFI has a moderate heritability (h^2^ = 0.29−0.46) in cattle, which makes it a good candidate for genetic improvement through selective breeding [1,8,9].

However, because RFI measurements are expensive and time-consuming, they are performed only on a small percentage of the cattle population. Simpler or cheaper proxies for measuring RFI are clearly desirable. Because RFI is a measure of metabolic efficiency, it has been proposed that metabolomics or metabolite measurements of bovine biofluids may offer a lower cost alternative to manual RFI measurement. Several metabolomics studies have been conducted in beef cattle to explore the relationship between RFI and metabolite levels [10,11,12]. For example, higher concentrations of glucose [10], urea [10], creatine [11], carnitine [11], and β-hydroxybutyrate [12], but lower concentrations of creatinine [10] were reported in the plasma of HRFI beef cattle as compared with LRFI beef cattle. However, neither the performance of these biomarkers nor a precise mathematical model for predicting RFI from these biomarkers has been described. Likewise, these studies were limited to measuring a relatively small number of metabolites via a single metabolomics platform (such as nuclear magnetic resonance (NMR) spectroscopy) or a laboratory chemistry analyzer. Here, we describe a more comprehensive metabolomic study that uses multiple metabolomics platforms, including NMR spectroscopy, liquid chromatography-tandem mass spectrometry (LC-MS/MS), and inductively coupled plasma mass spectrometry (ICP-MS), to quantitatively characterize 145 serum metabolites in HRFI and LRFI young Angus bulls. Using this comprehensive metabolomics dataset, we were able to identify several new metabolite biomarkers for RFI. Furthermore, we have constructed two logistic regression models (one optimized for NMR, the other optimized for LC-MS/MS) that use just two serum metabolites to differentiate HRFI and LRFI animals with a high sensitivity and specificity (AUROC > 0.85). A more complete description of the methods, the biomarkers, and the models are given below.

## 2. Results

### 2.1. The Serum Metabolome of Beef Cattle

Serum metabolomic data were obtained from 15 HRFI and 10 LRFI young Angus bulls using three metabolomics platforms including NMR, LC-MS/MS, and ICP-MS. A total of 145 metabolites were identified and quantified in each serum sample (Table 1). We have deposited this information into the Bovine Metabolome Database (BMDB) (www.bovinedb.ca) [13]. Inspection of our experimental data reveals that the chemical composition of bovine serum is dominated by inorganic ions (primarily sodium, potassium, calcium, and phosphorus), carbohydrates (glucose), organic acids (lactate, acetate, and 3-hydroxybutyrate), amino acids (glycine, valine, and glutamine), and various amine-containing compounds (urea, creatinine). We found that for those metabolites that were measured by both LC-MS/MS and NMR, there was a generally good overall agreement with the concentration values across both platforms. Therefore, to simplify the presentation of the data we only report the LC-MS/MS values for those metabolites measured on both platforms. According to our data, the range of metabolite concentrations detected in bovine serum varied from 1.2 ± 0.2 µM (fumarate) to 5393 ± 2341 µM (lactate) for NMR, from 0.0075 ± 0.0011 µM (C14:2-OH (hydroxytetradecadienylcarnitine)) to 4115 ± 326 µM (glucose) for LC-MS/MS, from 0.0016 ± 0.0001 µM (cesium) to 132,919 ± 3122 µM (sodium) for ICP-MS.

Using a combination of NMR and LC-MS/MS, a total of 58 water-soluble organic compounds were identified and quantified in bovine serum. The most abundant water-soluble organic compounds in serum were lactate (5393 ± 2341 µM), glucose (4115 ± 326 µM), and urea (1389 ± 266 µM). The lowest concentration that could be reliably detected in serum was 0.035 ± 0.021 µM for putrescine.

The TMIC Prime assay (a locally developed LC-MS/MS assay) provided quantitative results for 74 lipids or lipid-like compounds including 10 phosphatidylcholines (PCs), 14 lysophosphatidylcholines (LysoPCs), 5 sphingomyelins (SMs), 5 hydroxysphingomyelins (SM(OH)s), and 40 acylcarnitines (ACs) in bovine serum. Note that some LysoPC and PC species identified by the TMIC Prime assay correspond to multiple (ranging from as few as 2 to as many as 24) possible unique lipid structures. In our study, SM(16:0) (69 ± 10 µM) and C14:2-OH (hydroxytetradecadienylcarnitine) (7.5 ± 1.1 nM) were the most and least abundant lipid-like compounds identified in serum, respectively.

ICP-MS also provided quantitative results for 13 trace minerals in bovine serum. The most abundant elements identified and quantified by ICP-MS were sodium (134 ± 16 mM), potassium (4.3 ± 0.3 mM), calcium (2.2 ± 0.2 mM), and phosphorus (1.3 ± 0.2 mM). While the least abundant metals quantified by ICP-MS were cesium (1.6 ± 0.2 nM), barium (190 ± 40 nM), and strontium (940 ± 140 nM).

### 2.2. Univariate Statistical Analysis of Bovine Serum Metabolites

**Figure 1:**
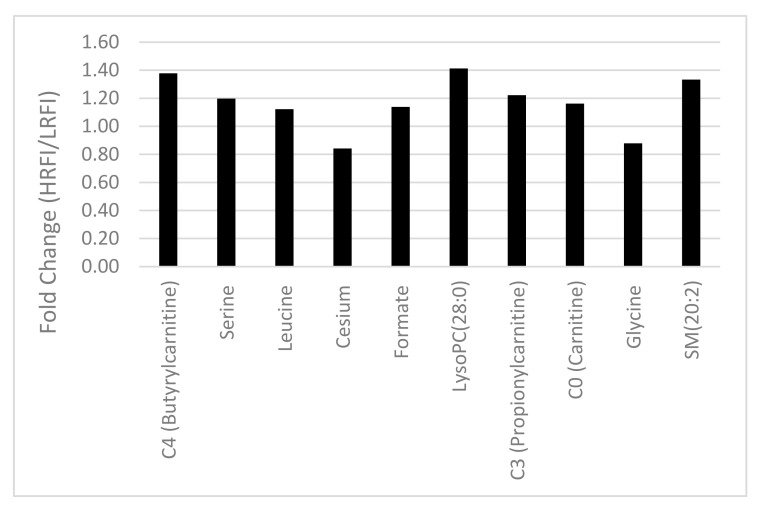
Comparison of fold change of significantly regulated metabolites (*p*-value < 0.05) in the serum of high-residual feed intake (HRFI) versus low-residual feed intake (LRFI) bulls.

Using univariate analysis, we compared the serum metabolite profile of those young Angus bulls identified as being HRFI with those identified as being LRFI. The most significantly different metabolites (*p*-value < 0.05) between the HRFI and the LRFI animals are shown in Figure 1. In total, 10 differentially expressed metabolites achieved statistical significance in this comparison. Specifically, the serum concentrations of serine, leucine, formate, C0 (carnitine), C3 (propionylcarnitine), C4 (butyrylcarnitine), LysoPC(28:0), and SM(20:2) were greater in HRFI bulls than LRFI bulls. The most upregulated metabolites were LysoPC(28:0) with a fold change (HRFI/LRFI) of 1.41 and C4 (butyrylcarnitine) with a fold change (HRFI/LRFI) of 1.38. In addition to these eight upregulated metabolites, two other metabolites, glycine and cesium, were downregulated in the HRFI bulls as compared with their LRFI counterparts.

### 2.3. Multivariate Analysis of Bovine Serum Metabolites

**Figure 2:**
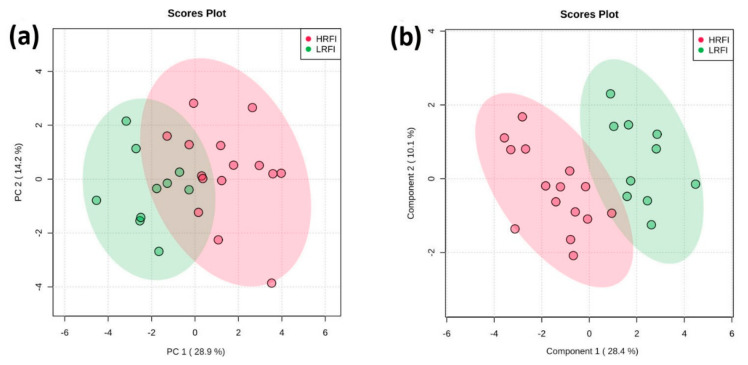
Comparison between serum metabolite data acquired for HRFI versus LRFI group. (**a**) Principal component analysis (PCA) graph; (**b**) Partial least squares discriminant analysis (PLS-DA) graph with permutation test *p*-value of < 0.01.

**Figure 3:**
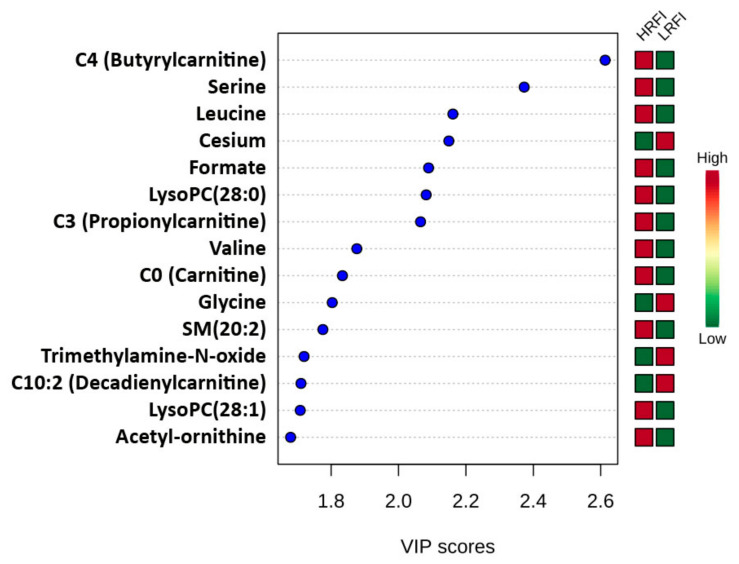
Variable importance in projection (VIP) plot acquired from the comparison between HRFI vs. LRFI group. The most discriminating metabolites are shown in descending order of their coefficient scores. The color boxes indicate whether metabolite concentration is increased (red) or decreased (green) in HRFI vs. LRFI group.

Principle component analysis (PCA) showed moderately separable clustering between HRFI and LRFI animals (Figure 2a), while partial least squares discriminant analysis (PLS-DA) showed a good separation for these two groups (Figure 2b). Permutation tests conducted on the PLS-DA model indicated that the observed separation was statistically significant (*p*-value < 0.01). A variable importance of projection (VIP) plot of the PLS-DA data, which ranks the top 15 metabolites based on their contribution to the discriminant model, is shown in Figure 3. The heat map on the right side of the VIP plot indicates that four metabolites (cesium, glycine, trimethylamine-N-oxide, and C10:2 (decadienylcarnitine)) were more abundant in the LRFI group, while the other 11 metabolites were more abundant in the HRFI group. All, except five metabolites (valine, trimethylamine-N-oxide, C10:2 (decadienylcarnitine), LysoPC(28:1), and acetyl-ornithine), identified via our multivariate analysis overlapped with the metabolites identified as significantly different between LRFI and HRFI animals by our univariate analysis.

### 2.4. Biomarkers for Bovine RFI

From the significant metabolites identified via our univariate and multivariate analyses, we used logistic regression to generate two optimal models for distinguishing HRFI from LRFI animals. One biomarker panel uses only NMR-acquired data while the second uses only LC-MS/MS acquired data. The NMR model used two metabolites that are easily measured by NMR, i.e., formate and leucine (with an AUROC of 0.92 and a *p*-value of < 0.01). The LC-MS/MS model also used two metabolites that are easily measured by LC-MS/MS, i.e., C4 (butyrylcarnitine) and LysoPC(28:0) (with an AUROC of 0.89 and a *p*-value of < 0.01).

**Figure 4:**
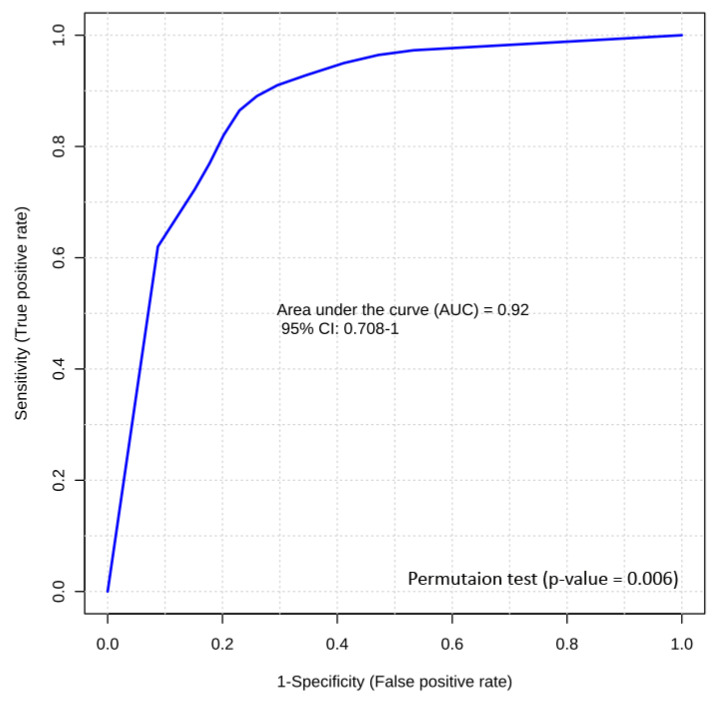
Biomarker analysis of bovine RFI. Logistic regression receiver operating characteristic (ROC) curve analysis of a panel of two NMR-detectable candidate biomarkers (formate and leucine) from bovine serum samples.

As noted above, the best performing panel was the NMR-based test, which included formate and leucine. A logistic regression equation for these two candidate biomarkers was used to calculate the receiver operating characteristic (ROC) curve and to calculate the area under the ROC curve or AUROC (Figure 4). Permutation testing (*n* = 1000) confirmed the significance of this model (*p*-value = 0.006). The logistic regression model developed for this prediction is given as follows:

logit(P) = log(P/(1 − P)) = −1.6 − 3.554 × formate − 2.161 × leucine(1)

where P is the probability of an animal being classified as LRFI. The optimal cutoff point for the above equation is 0.38. This means that an animal with a value greater than or equal to 0.38 belongs to the LRFI group, while an animal with a value less than 0.38 belongs to the HRFI group. Because the concentrations of the metabolites used in this study were cube-root transformed, and then scaled via auto scaling, the value for formate in the above equation corresponds to the (cube root [formate] − 4.2684)/0.1951 (where [formate] is the measured concentration of this compound in µM, as quantified by NMR). Likewise, the value for leucine corresponds to the (cube root [leucine] − 5.9522)/0.2257 (where [leucine] is the measured concentration of this compound in µM, as quantified by NMR).

**Figure 5:**
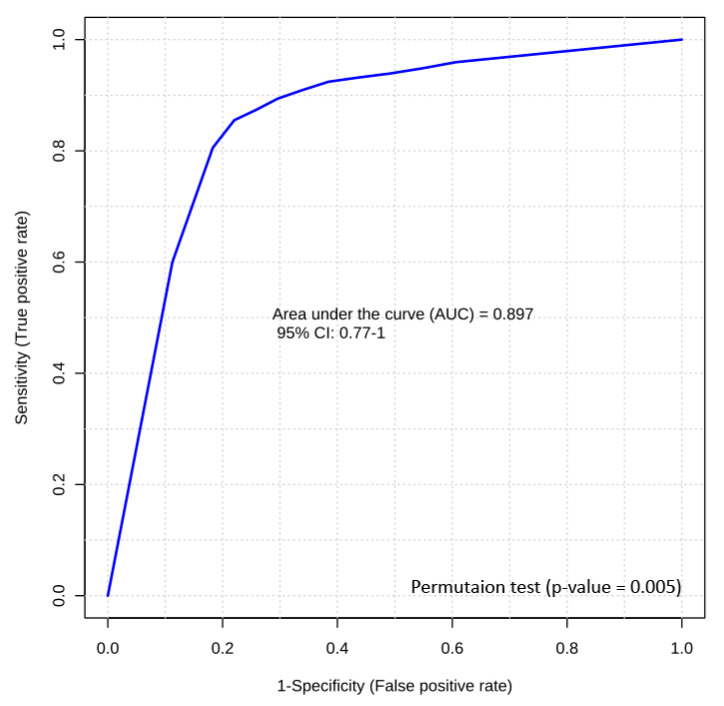
Biomarker analysis of bovine RFI. Logistic regression ROC curve analysis of a panel of two LC-MS/MS candidate biomarkers (C4 (butyrylcarnitine) and LysoPC(28:0)) from bovine serum samples yields an AUC of 0.89.

The second best performing RFI prediction panel included two metabolites that could only be measured by LC-MS/MS, i.e., C4 (butyrylcarnitine) and LysoPC(28:0). A logistic regression equation for these two candidate biomarkers was used to generate a model with a final AUROC of 0.89 (Figure 5). Permutation testing (*n* = 1000) confirmed its significance (*p*-value = 0.005). The logistic regression model developed for this prediction is given as follows:

logit(P) = log(P/(1 − P)) = −3.625 − 5.351 C4(butyrylcarnitine) − 6.378 LysoPC(28:0)(2)

where P is the probability of an animal being classified as LRFI. The optimal cutoff point for the above equation is 0.53. This means that an animal with a value greater than or equal to 0.53 belongs to the LRFI group, while an animal with a value less than 0.53 belongs to the HRFI group. Because the concentrations of the metabolites used in this study were cube root transformed, and then scaled via auto scaling method, the value for C4 (butyrylcarnitine) in the above equation corresponds to the (cube root [C4(butyrylcarnitine)] − 0.5557)/0.0499 (where [C4(butyrylcarnitine)] is the measured concentration of this compound in µM, as quantified by LC-MS/MS). Likewise, the value for LysoPC(28:0) corresponds to the (cube root [LysoPC(28:0)] − 0.6494)/0.0726 (where [LysoPC(28:0)] is the measured concentration of this compound in µM, as quantified by LC-MS/MS).

## 3. Discussion

The main objective of this study was to identify candidate serum biomarker metabolites that could successfully discriminate HRFI cattle from LRFI cattle. To optimize the likelihood of identifying robust RFI biomarkers we used a combination of three quantitative metabolomics platforms (NMR, LC-MS/MS, and ICP-MS). Using these three platforms, we were able to identify and quantify a total of 145 metabolites, including 58 water-soluble organic compounds, 74 lipid-like compounds, as well as 13 metal ions. Overall, we found a very good agreement between the results of these 145 experimentally quantified metabolites with those of reported elsewhere (available in www.bovinedb.ca [13]). Indeed, the concentrations reported for serum in the BMDB agreed well with our experimental data. For instance, the value of asparagine reported by our study ranged from 21 to 30 µM, and for the literature-derived data it ranged from 20 to 33 µM. This widespread agreement was not unexpected, because serum/plasma must be highly stable and cannot vary much in its metabolite concentrations, to ensure physiological homeostasis [14].

Of course, there were a few exceptions to this rule. The most variable metabolite reported in serum was betaine. The value of betaine reported by our study ranged from 131 to 205 µM, and the literature-reported values ranged from 14 to 26 µM [15]. This variation could be due to a number of factors, including differences in diet, sex, age, breed, sample work-up or extraction, sample storage protocols, analytical platforms, and instrument sensitivity. We believe the most likely contributor to this difference is diet, as the amount of betaine in the diet of our beef cattle would be expected to be different than that of dairy cattle in the reference study of Artegoitia et al. [15]. Overall, there were very few outliers like betaine. Therefore, the good agreement for metabolite concentrations we obtained for the Angus bulls used in this study, with other cattle breeds suggests that the RFI biomarkers we discovered here should be transferrable to other breeds of beef cattle fed similar kinds of diets.

### 3.1. Comparison with Literature-Reported Biomarkers of Bovine RFI

To date, there have been four other published metabolomic studies that have attempted to identify relationships between blood metabolite levels and bovine RFI [10,11,16,17]. The study of Fitzsimons et al. [10] showed higher concentrations of glucose and urea and lower concentrations of creatinine in the plasma of HRFI vs. LRFI heifers. The study by Karisa et al. [11] reported higher concentrations of creatine, carnitine, formate, hydroxyisobutyrate, and tyrosine in the plasma of HRFI beef cattle along with higher concentrations of glycine in the plasma of LRFI beef cattle. Clemmons et al. [16] reported that the serum concentrations of pantothenate, homocysteine, glutamine, and carnitine were found to be associated with divergent RFI in beef steers, although no concentration values for these metabolites were reported in the Clemmons et al. study. A very recent study conducted by Jorge-Smeding et al. [17] found that plasma metabolites that are directly (ornithine) or indirectly (aspartate, lysine, valine) associated with the urea cycle were correlated with RFI in Charolais heifers.

Table 2 summarizes these previous metabolomic findings and compares them with the findings reported here. As can be seen in this table, there was a good agreement between our findings and those reported from Fitzsimons et al. [10], Karisa et al. [11], and Jorge-Smeding [17]. For example, serum/plasma concentrations of tyrosine were higher in our HRFI group, which is in agreement with the findings of Fitzsimons et al. [10], and Karisa et al. [11]. Likewise, the serum concentrations of valine was higher in the HRFI group, which is similar to the findings of Fitzsimons et al. [10] and Jorge-Smeding et al. [17]. However, there were some discrepancies, with the most significant variations being seen in the study of Karisa et al. [11]. For instance, the serum concentration of 3-hydroxybutyrate was higher in HRFI animals in our study but reported as being lower in the study of Karisa et al. [11]. Karisa et al. [11] also reported exceptionally high concentrations for succinate (~250 µM), oxobutyrate (~40 µM), and allantonin (~90 µM), which do not match values reported by our study, by any other bovine studies, or by the referential data in the BMDB [13]. Indeed, closer analysis of the NMR spectral regions corresponding to these metabolites (especially at higher fields) suggests that these peaks may have been incorrectly identified, and therefore incorrectly quantified. Other reasons for the differences between the Karisa et al. study and other bovine studies could be due to differences in diet, sex, age, breed, sample work-up or extraction, or instrument sensitivity.

Another notable difference was found for blood glucose concentrations between our study and the values reported by Fitzsimons et al. [10]. In particular, the concentration of glucose was found to be higher in the serum of LRFI Angus bulls in our study but reported as being higher in the plasma of medium- and high-RFI Simmental heifers, respectively, in the study of Fitzsimons et al. [10]. Apart from glucose, other metabolites measured in both studies showed similar trends in terms of RFI classification (i.e., both studies found that the concentration of urea and carnitine were higher in HRFI animals). Glucose concentrations can vary significantly depending on how long samples are left at room temperature prior to being frozen. This is because glycolytic reactions in liquid serum/plasma can readily lead to conversion of glucose to lactate. Unfortunately, no details were provided in the study by Fitzsimons et al. [10] regarding sample preparation time or lactate levels. Furthermore, given the fact that the highest concentration of glucose was seen in medium-RFI animals as opposed to the LRFI or HRFI animals, suggests the glucose data reported by Fitzsimons et al. may have been more reflective of differences in sample preparation time than true differences in RFI. As a general rule, we treat reported glucose concentrations in livestock studies with a good deal of caution because of the extreme sensitivity of glucose levels to sample preparation/storage.

### 3.2. Candidate Serum Biomarkers of Bovine RFI

While other studies have identified possible associations between blood metabolites and bovine RFI, as yet, no published study has attempted to develop quantitative metabolite biomarker panels to predict RFI in cattle. Using logistic regression models, two categorical predictive biomarker panels were developed from this study to categorically predict RFI and to distinguish HRFI animals from LRFI animals.

The best performing panel was an NMR-based, two-metabolite model that included formate and leucine. The second-best performing panel was an LC-MS/MS based two-metabolite model that included C4 (butyrylcarnitine) and LysoPC (28:0). Both panels have high sensitivity and specificity (AUROC > 0.85), making them good candidates to distinguish or predict HRFI animals from LRFI animals. Because these panels consist of just two metabolites, it is possible to construct very fast (<5 min/sample) and inexpensive (<$10) NMR or MS-based assays that could be used to perform bovine RFI characterization.

Basarab et al. [18] has estimated that a mature LRFI cow would have a net economic profit of $46/head/year as compared with that of HRFI. This cost calculation suggests that selecting for LRFI could have a significant effect on reducing the costs of production for cattle ranchers. However, RFI is a difficult and time-consuming measure to perform. The cost of performing RFI measurements over 80–90 days is ~ $250/head which is much higher than the cost of a metabolite test ($5–10/head) or the net profit of selecting for LRFI cattle via GrowSafe^™^ RFI measurements. Therefore, a simple blood test that could distinguish high RFI (HRFI) animals from LRFI animals (early on) would potentially benefit beef farmers in terms of optimizing production or selecting which animals to cull or which animals should be bred.

As noted in the Methods section, the serum samples used to perform these metabolomic assays were collected at 15 months (shortly before the cattle were slaughtered at 17 months). Beef cattle produced in the United States and Canada can be slaughtered at any time from 12 months to 24 month of age, with the highest quality beef coming from those slaughtered under 24 months of age and the most tender meat found in animals slaughtered between 12–18 months of age [19]. Therefore, the markers identified here could be used for reasonably early prediction of RFI performance. However, it is not clear if the same panel of metabolites would work at other ages (14 months, 12 months, or 9 months) or whether the same panel would also work with cows, steers or heifers. Other metabolomic studies that have looked at metabolite-RFI associations [20,21] suggest that these metabolic traits are likely established early in an animal’s lifetime, and so there is a good likelihood that these biomarkers could be used to assess RFI earlier than 15 months. Being able to perform a serum-based RFI “prediction”, even earlier in an animal’s lifetime, would certainly allow critical decisions to be made by the producer regarding breeding, culling, or feeding a particular animal.

It is also important to note that other physiological factors certainly play a role in the composition of the bovine metabolome (and therefore the biomarker panel parameters described here), including physical maturity, sex, and castration status. These physiological and age-dependent differences would be expected to lead to changes in the optimal cut-off concentrations. Typically, bulls are castrated at three to six weeks of age to become steers [22]. Uncastrated bulls reach puberty at 9–10 months [23], while heifers reach puberty at 12–14 months [24]. Castration certainly affects some aspects of bovine metabolism as does the stage of an animal’s sexual and physical maturity. Clearly, additional studies need to be done with other bovine cohorts over a range of ages and a range of physiological states to confirm the utility and cut-off values for these RFI biomarkers.

In addition to working with animals covering a wider set of ages (to ascertain the RFI biomarker age-range), it would also have been useful to perform further validation of these biomarkers on a “hold out” set of animals. These hold-out animals would have ideally raised elsewhere or at a different time using similar feeding, housing, and animal management conditions. However, the high costs of measuring RFI, the length of the study (almost two years) and the costs of maintaining the animals for 17 months make these sorts of studies prohibitively expensive, especially given the limited resources for this sort of discovery-based study.

### 3.3. Metabolite Markers and Their Role in RFI Biochemistry

Our study identified a number of significantly different metabolites that seemed to drive the observed differences in RFI, i.e., C4 (butyrylcarnitine), LysoPC(28:0), formate, and leucine. Each of these compounds plays an important role in bovine metabolism. C4 (butyrylcarnitine) is an acylcarnitine formed when fatty acyl-coenzyme A (fatty acyl-CoA) enters through the carnitine shuttle into the mitochondria for β-oxidation and the tricarboxylic acid (TCA) cycle to produce ATP [25]. Besides facilitating fatty acids crossing the mitochondrial membranes to be degraded by β-oxidation, acylcarnitines along with branched-chain amino acids (BCAAs) (leucine, iso-leucine, and valine), also mediate activation of several important hepatic metabolic signaling pathways leading to diseases such as non-alcoholic fatty liver disease and type 2 diabetes mellitus in humans and other mammals [26,27]. The short-chain acylcarnitines C3, C4, and C5 are degradation products of BCAAs [28] and saturation of the BCAA degradation pathway has been shown to inhibit the initial step of β-oxidation, leading to weight gain and body fat deposition [28]. High concentrations of BCAAs are associated with higher oxidative stress, and as seen in human and rodent studies, can serve as biomarkers for obesity-associated insulin resistance and diabetes [28,29].

LysoPC(28:0) belongs to the lysophosphatidylcholine family of lipids which are derived by partial hydrolysis of phosphatidylcholines by removing one of the fatty acid groups, via the action of phospholipase A2 (PLA2) [30]. High concentrations of lysoPC species (especially those containing palmitoyl (C16:0) or stearoyl (C18:0) groups) in the blood are known to stimulate cytosolic PLA2 and this results in an increased release of arachidonate, which is associated with cardiovascular disease [31]. In the vascular system, lysophosphatidylcholines have been shown to increase oxidative stress [32,33,34]. For example, Zou et al. [34] reported that lysophosphatidylcholines enhanced oxidative stress in rat aorta during aging via the 5-lipoxygenase pathway. Lehmann et al. [35] reported that circulating lysophosphatidylcholines can serve as biomarkers of a metabolically benign non-alcoholic fatty liver in humans. In particular, Lehmann et al. found that the plasma concentration of lysophosphatidylcholines was higher in insulin-sensitive patients with non-alcoholic fatty liver as compared with insulin-resistant ones with non-alcoholic fatty liver. Stiuso et al. [36] also reported that lipidomic or oxidative status of serum caused by lysophosphatidylcholines was associated with liver diseases (i.e., non-alcoholic fatty liver or steatohepatitis). A recent study [37] reported lower levels of reactive oxygen species (ROS) in the liver of LRFI steers which suggested they have lower levels of hepatic oxidative stress than HRFI steers. Decreased oxidative stress in the liver has been associated with lower feed maintenance requirements, due to a lower lipid and protein turnover and better efficiency in energy usage [37].

As comprehensively reviewed by Herd and Arthur [38], variations in RFI can be explained by differences in energy expenditure from metabolic processes, body composition, and physical activity. Typically, greater energy expenditures and higher maintenance requirements are seen in HRFI animals as compared with LRFI animals [38]. Richardson et al. [39] also reported that Angus steers born from HRFI parents had less whole-body protein and more whole-body fat as compared with progeny steers of LRFI parents. Therefore, higher levels of C4 (butyrylcarnitine) and LysoPC(28:0) in the serum of our HRFI bulls might be associated with increased oxidative stress in the HRFI group.

The other two serum metabolites that were most differentiating between HRFI and LRFI animals included formate and leucine. The concentration of formate and leucine was higher in HRFI animals and lower in LRFI animals. Formate participates in NADPH synthesis and catalyzes the conversion of fumarate into succinate in the TCA cycle [40,41]. The associations between RFI and several metabolites (i.e., acetate, citrate, and succinate) linked to the TCA cycle were recently discussed by Karisa et al. [11], as well as Wang and Kadarmideen [42]. Formate is the simplest carboxylic acid and serves as a potent reductive force against oxidative stress. It is produced when the keto-acid, glyoxylate, neutralizes ROS in cells [40,41]. Therefore, a higher level of formate in the serum of HRFI animals suggests that these less feed-efficient cattle are more prone to oxidative stress in the form of higher levels of ROS. This conclusion also agrees with the results reported in the study performed by Casal et al. [37]. Additionally, Fitzsimons et al. [10] also reported positive correlations between RFI and formate levels in the plasma of beef cattle.

Leucine is a branched-chain amino acid and its catabolism generates succinyl-CoA and acetyl-CoA, both of which can upregulate the activity of the TCA cycle [43,44]. BCAAs are also involved in protein turnover in skeletal muscle [44,45,46]. Leucine also increases fatty acid oxidation [47]. As discussed earlier, high concentrations of BCAAs are associated with higher levels of oxidative stress [28,29]. Therefore, leucine could have an important role in RFI variation, since both protein turnover, oxidative stress, and energy metabolism are key factors affecting this phenotype [38].

We also performed a further study to understand if the variations in the concentration of C4 (butyrylcarnitine), LysoPC(28:0), formate, and leucine between HRFI and LRFI bulls correlated with the concentration of these metabolites in their rumen. This was done to explore whether these metabolite difference may be associated with differences in ruminal activity or rumen microbial activity. However, we found no such correlation (data not shown).

## 4. Materials and Methods

### 4.1. Ethics Approvals

The collection and analysis of bovine serum in this study was approved by the University of Alberta’s Animal Care Committee (Animal Use Protocol [AUP] 1129) under the auspices of the Canadian Council of Animal Care [48].

### 4.2. Animals and Experimental Design

Twenty-five purebred Angus bulls, raised on the University of Alberta’s Roy Berg Kinsella Research Ranch (Kinsella, AB, Canada), were used in this study. After weaning, bulls were fed and managed according to industry standards for production of potential replacement yearling bulls in Alberta until their RFI test at approximately 13 months of age [49].

### 4.3. Measurement of Phenotypic RFI Values for the Angus Bull Cohort

From the end of May 2015 until mid-August 2015, bulls were tested for RFIf (RFI that was adjusted for rib fat thickness at the end of feedlot test) at approximately 13 to 16 months of age using the GrowSafe^™^ automated feed recording system (GrowSafe Systems Ltd., Calgary, AB, Canada) at Agriculture and Agri-Food Canada (AAFC, Lacombe, AB, Canada). The RFI test was conducted following the protocols and calculation of RFI as reported by Mao et al. [50] and Johnson et al. [49], except that standardized daily dry matter intake (STDDMI) was calculated as an average of dry matter intake over the test period and standardized to 12 megajoules of metabolizable energy (MJ ME) per kg dry matter for finishing bulls (instead of 10 MJ ME for heifers). The GrowSafe diet consisted of 45% barley and 55% silage (as fed basis), and the nutrient analysis is presented in Table 3. An adaptation period of 21 days was used to acclimatize cattle to the GrowSafe system and diet. The quantity of feed intake for each feeding event of each bull was recorded by the GrowSafe system, which was further used to calculate total feed intake over the 77-day test period. Bulls were weighed twice at the beginning of test, once per month throughout the test, and once at slaughter, which was a few days after the RFI test was complete.

The end of the RFI test weight was estimated from the slaughter weight. Rib fat thickness measurements (12/13th rib fat depth and LT area) were also determined at end of test, using an Aloka SSD-210 portable ultrasonographic scanner (Aloka Co., Tokyo, Japan). The initial BW at the start of the test and ADG were derived from a linear regression of the serial BW measurements against time (day). Then, the metabolic BW (MWT) in kg was calculated as midpoint BW^0.75^, where the midpoint BW was computed as the sum of the initial BW and the product of ADG multiplied by half of the days on test. Using the dry matter (DM) content of the diet, as well as the bull’s daily intake, daily DMI in kg was calculated as an average of dry matter intake over the test period and was further standardized to 12 MJ ME per kg dry matter (STDDMI). In order to generate regression coefficients to predict an animal’s expected DMI required for maintenance of body weight and growth, a linear regression model was fit using PROC GLM in SAS (SAS Institute, Inc., Cary, NC, USA). The model was as follows:

Y*_i_* = β_0_ + β_1_ADG*_i_* + β_2_MWT*_j_* + β_3_FUFAT*_k_* + e*_ijk_* {1} (3)

where Y*_i_* is the STDDMI for the *i*th bull, β_0_ is the intercept, β_1_ is the partial linear regression coefficient of ADG, β2 is the partial linear regression coefficient of MWT, β_3_ is the partial regression coefficient of final ultrasound backfat thickness (FUFAT), and e*_i_* is residual error for the *i*th bull. RFIf in kg of DMI per day (kg/day) was computed as the difference between the standardized daily DMI and the expected DMI that was predicted based on animal’s ADG, MWT, and ultrasound backfat thickness in mm at the end of feedlot test (FUFAT) using the regression intercept and regression coefficients resulting from {1}. In total, 15 HRFI (0.39 ± 0.28 (mean RFIf ± standard deviation (SD))) and 10 LRFI (−0.52 ± 0.26) bulls were identified in this study. Those animals with RFIf value higher and lower than 0 kg/day were classified as HRFI and LRFI, respectively. RFIf values ranged from −1.05 kg to +1.07 kg DM per day, with an average of 0.00 kg/day.

### 4.4. Sample Collection

Blood samples (10 mL) were collected in the morning (just before feeding) at 15 months of age from a jugular vein using vacutainer serum collection tubes (Becton Dickinson, Mississauga, ON, Canada). Blood samples were kept in a cooler on ice, transferred to the laboratory within 3 h after collection, and centrifuged at 2000× *g* at 4 °C for 15 min. Then, the upper layer of serum was collected, and 4 mL was stored at −80 °C.

### 4.5. Metabolomics Tests

Three metabolomics platforms, including NMR, LC-MS/MS, and ICP-MS, were used to identify and quantify a total of 145 metabolites in each bovine serum sample. Using NMR, LC-MS/MS, and ICP-MS, 42, 116, and 13 metabolites were identified and quantified, respectively, of which 26 metabolites were common between NMR and LC-MS/MS. Details of sample preparation along with how the samples were run on each metabolomics platform have been previously described in detail by Foroutan et al. [13,51]. Briefly, for NMR analysis, serum samples were filtered using a 3-kDa ultrafiltration unit (Amicon Micoron YM-3, Sigma-Aldrich, St. Louis, MO, USA) to remove large molecular weight proteins and lipoproteins. These macromolecules can seriously compromise the quality of ^1^H-NMR spectra though the generation of intense, broad lines that interfere with the identification and quantification of lower abundance metabolites [51]. Then, the de-proteinized sample was frozen and stored at −80 °C until further use. For NMR spectroscopic analysis, 280 μL of the ultra-filtered serum was transferred to a 1.5 mL Eppendorf tube, to which an additional 70 μL of a standard NMR buffer solution (250 mM potassium phosphate (pH 7.0), 5 mM 2,2-dimethyl-2-silapentane-5 sulfonate (DSS-d_6_), 5.84 mM 2-chloropyrimidine-5-carboxylic acid, and D_2_O 54% *v/v* in H_2_O) was added. The mixture was then transferred to a 3 mm NMR tube for spectral analysis. All ^1^H-NMR spectra were collected on a Bruker Avance III Ascend 700 MHz spectrometer equipped with a 5 mm cryo-probe (Bruker Biospin, Rheinstetten, Germany). Compound identification and quantification by NMR were performed according the procedure described by Foroutan et al. [52], using the Chenomx NMR Suite 8.1 software package (Chenomx Inc., Edmonton, AB, Canada).

A targeted, quantitative LC-MS/MS metabolite profiling approach was employed that combined reverse-phase liquid chromatography and mass spectrometry (RPLC-MS) with direct flow injection (DFI) mass spectrometry (DFI-MS) (RPLC-DFI-MS/MS). LC-MS/MS was employed to determine the concentrations of up to 143 compounds (including amino acids, biogenic amines, glucose, organic acids, acylcarnitines, PCs, LysoPCs, SMs, and SM(OH)s) using an in-house quantitative metabolomics assay (TMIC Prime) [13,53,54]. All LC-MS analyses were conducted on an AB SCIEX QTRAP^®^ 4000 mass spectrometer (Sciex Canada, Concord, ON, Canada) equipped with an Agilent 1260 series UHPLC system (Agilent Technologies, Palo Alto, CA, USA). The Analyst software 1.6.2 (Concord, ON, Canada) was used to control the entire assay’s workflow. The macro- and micro-elemental analyses were performed on a NexION 350× ICP-MS (Perkin Elmer, Woodbridge, ON, Canada) according the procedure described by Foroutan et al. [13].

### 4.6. Statistical Analysis

Data analysis was performed using MetaboAnalyst 4.0 according to previously published protocols [55,56]. Those metabolites having more than two missing values in each group were removed from further analyses. A univariate analysis including *t*-tests and fold-change analysis was performed in order to identify differentially expressed metabolites between the HRFI and LRFI groups. Statistical significance was declared at a *p*-value < 0.05.

Multivariate statistics, including PCA, PLS-DA, and ROC curve analysis, were performed using MetaboAnalyst 4.0. The data was scaled and normalized using a cube root transformation and auto scaling, which generated a clear Gaussian distribution plot prior to multivariate analysis. A permutation test involving 2000 randomized datasets was implemented to minimize the possibility that the observed separation of the PLS-DA was due to chance (a valid model should have a *p*-value < 0.05).

ROC curves were calculated by MetaboAnalyst 4.0 to evaluate the predictive ability of potential metabolic biomarkers using a logistic regression model. The area under the ROC curve (AUC or AUROC) was used to interpret the performance across the two different biomarker models to determine the best cut-off point for maximal sensitivity and specificity. A ROC curve plots the false-positive rate (1-specificity) on the X axis versus sensitivity on the Y axis. On the one hand, sensitivity (or recall) is defined as the number of true positives divided by the sum of the true positives and false negatives. On the other hand, specificity is defined as the number of true negatives divided by the sum of the true negatives and false positives. In a ROC curve, the accuracy of a test for correctly distinguishing one group from another, such as HRFI bulls from LRFI bulls, is measured by the area under the ROC curve (AUROC). The AUROC equal to 1 is the highest value indicating a perfect discriminating test, which is obtained when all positive samples are ranked before negative ones. A permutation test involving 1000 randomized permutations was implemented to validate (a valid model should have a *p*-value < 0.05) the reliability of the model for each ROC curve.

## 5. Conclusions

In this study we evaluated the effectiveness of using multi-platform, quantitative metabolomics to identify candidate serum biomarkers that can easily distinguish HRFI animals from LRFI animals. LC-MS/MS, NMR, and ICP-MS were used to identify and quantify 145 serum metabolites in an effort to maximize our chances to identify and develop a suitable set of metabolite RFI biomarkers. We successfully identified two significant candidate biomarkers panels (AUROC > 0.85) that can predict RFI categorically. These include a two-metabolite model (formate and leucine) that is compatible with NMR analysis and a two-metabolite model (C4 (butyrylcarnitine) and LysoPC(28:0)) that is compatible with LC-MS/MS analysis. These results suggest that serum metabolites could be used to categorically predict RFI (early on) and inexpensively distinguish HRFI cattle from LRFI cattle.

While the results we obtained are very statistically significant and appear to be consistent with other reported studies on bovine RFI, the main limitation in this study was the small sample size (15 HRFI vs. 10 LRFI cattle). Given the significant costs and time associated with performing RFI measurements on cattle, this is a limitation that is difficult to overcome. Another limitation lies in the fact that the study was conducted on only a single sex (bulls), from a single breed (Angus cattle), consuming the same diet. However, it is important to note that we demonstrated that the data we measured in this study was broadly consistent with data collected for other beef cattle RFI studies. This gives us reason to believe that the results presented here will be shown to be largely reproducible elsewhere. Nevertheless, in order to properly confirm the robustness of these serum biomarkers as proxies to distinguish between divergent RFI cattle, further validation studies using a larger cohort of cattle with more diverse genetic backgrounds and from different management settings will be needed.

## Figures and Tables

**Table 1:** List of serum metabolites along with their analytical platform, measured concentrations, fold change, and log2 fold change.

Metabolite	Platform	HRFI (µM)	LRFI (µM)	Fold Change (HRFI/LRFI)	Log2 Fold Change (HRFI/LRFI)
**AMINO ACIDS**					
Alanine	LC-MS/MS & NMR	236 ± 29 ^1^	245 ± 30	0.96	−0.05
Arginine	LC-MS/MS & NMR	218 ± 32	218 ± 35	1.00	0.00
Asparagine	LC-MS/MS & NMR	26 ± 4	24 ± 3	1.08	0.12
Aspartate	LC-MS/MS & NMR	26 ± 12	22 ± 10	1.18	0.24
Beta-alanine	NMR	8 ± 1	8 ± 1	0.99	−0.02
Citrulline	LC-MS/MS & NMR	93 ± 15	81 ± 14	1.15	0.20
Creatine	LC-MS/MS & NMR	194 ± 31	199 ± 23	0.97	−0.04
Glutamate	LC-MS/MS & NMR	93 ± 22	89 ± 15	1.04	0.06
Glutamine	LC-MS/MS & NMR	330 ± 52	330 ± 22	1.00	0.00
Glycine *	LC-MS/MS & NMR	377 ± 66	429 ± 52	0.88	−0.19
Histidine	LC-MS/MS	78 ± 12	79 ± 8	0.99	−0.02
Isoleucine	LC-MS/MS & NMR	156 ± 15	150 ± 11	1.04	0.06
Leucine *	LC-MS/MS & NMR	221 ± 25	197 ± 15	1.12	0.17
Lysine	LC-MS/MS & NMR	91 ± 18	84 ± 9	1.08	0.12
Methionine	LC-MS/MS & NMR	33 ± 5	34 ± 3	0.97	−0.04
Ornithine	LC-MS/MS & NMR	60 ± 13	63 ± 12	0.95	−0.07
Phenylalanine	LC-MS/MS & NMR	72 ± 7	69 ± 7	1.04	0.06
Proline	LC-MS/MS & NMR	105 ± 15	101 ± 16	1.04	0.06
Serine *	LC-MS/MS & NMR	91 ± 13	76 ± 10	1.20	0.26
Threonine	LC-MS/MS & NMR	76 ± 12	72 ± 13	1.06	0.08
Tryptophan	LC-MS/MS	47 ± 7	47 ± 6	1.00	0.00
Tyrosine	LC-MS/MS & NMR	91 ± 12	90 ± 6	1.01	0.02
Valine	LC-MS/MS & NMR	367 ± 33	338 ± 28	1.09	0.12
**BIOGENIC AMINES**					
Acetyl-ornithine	LC-MS/MS	3.3 ± 0.71	2.8 ± 0.74	1.18	0.24
Asymmetric-dimethylarginine	LC-MS/MS	1.15 ± 0.21	1.06 ± 0.11	1.08	0.12
Carnosine	LC-MS/MS	31 ± 16	29 ± 6	1.07	0.10
Creatinine	LC-MS/MS & NMR	109 ± 18	118 ± 16	0.92	−0.11
Kynurenine	LC-MS/MS	7.3 ± 1.2	7.6 ± 2.4	0.96	−0.06
Methionine-sulfoxide	LC-MS/MS	1.2 ± 0.3	1.2 ± 0.3	1.00	0.00
Methylhistidine	LC-MS/MS	15 ± 2	14 ± 2	1.07	0.10
Putrescine	LC-MS/MS	0.035 ± 0.021	0.041 ± 0.014	0.85	−0.23
Sarcosine	LC-MS/MS & NMR	2.79 ± 0.73	3.08 ± 0.74	0.90	−0.15
Serotonin	LC-MS/MS	8 ± 3	10 ± 4	0.80	−0.32
Spermidine	LC-MS/MS	0.21 ± 0.01	0.18 ± 0.01	1.17	0.22
Spermine	LC-MS/MS	0.21 ± 0.14	0.12 ± 0.04	1.75	0.81
Taurine	LC-MS/MS & NMR	80 ± 25	81 ± 10	0.99	−0.02
Total-dimethylarginine	LC-MS/MS	2.1 ± 0.3	2.1 ± 0.3	1.00	0.00
Trans-hydroxyproline	LC-MS/MS	24 ± 5	27 ± 4	0.89	−0.17
Trimethylamine-N-oxide	LC-MS/MS	5 ± 1	7 ± 4	0.71	−0.49
**CARBOHYDRATES**					
Glucose	LC-MS/MS & NMR	3860 ± 490	4115 ± 326	0.94	−0.09
**ORGANIC ACIDS**					
3-hydroxybutyrate	NMR	375 ± 164	287 ± 94	1.31	0.39
Acetate	NMR	452 ± 228	329 ± 123	1.37	0.46
Alpha-aminoadipate	LC-MS/MS	1.25 ± 0.54	1.31 ± 0.44	0.95	−0.07
Ascorbate (Vitamin C)	NMR	11 ± 3	10 ± 3	1.10	0.14
Formate *	NMR	82 ± 13	72 ± 3	1.14	0.19
Fumarate	NMR	1.2 ± 0.2	1.2 ± 0.2	1.00	0.00
Lactate	NMR	4488 ± 1761	5393 ± 2341	0.83	−0.27
Pyruvate	NMR	142 ± 27	162 ± 54	0.88	−0.19
**MISCELANEOUS**					
Acetone	NMR	71 ± 27	69 ± 12	1.03	0.04
Betaine	LC-MS/MS & NMR	169 ± 27	168 ± 37	1.01	0.01
Choline	LC-MS/MS & NMR	20 ± 4	22 ± 4	0.91	−0.14
Ethanol	NMR	7.8 ± 1.2	8.1 ± 1.4	0.96	−0.05
Glycerol	NMR	312 ± 41	318 ± 36	0.98	−0.03
Isopropanol	NMR	2.27 ± 0.82	2.54 ± 0.34	0.92	−0.12
Methanol	NMR	32 ± 5	31 ± 3	1.03	0.05
Myo-inositol	NMR	43 ± 12	48 ± 8	0.90	−0.16
Urea	NMR	1389 ± 266	1220 ± 289	1.14	0.19
Uridine	NMR	3.1 ± 0.71	2.8 ± 0.52	1.11	0.15
**PHOSPHATIDYLCHOLINES, ACYL-ALKYL**					
PC ae (36:0)	LC-MS/MS	1.68 ± 0.41	1.64 ± 0.41	1.02	0.03
PC ae (40:6)	LC-MS/MS	0.47 ± 0.13	0.44 ± 0.04	1.07	0.10
**PHOSPHATIDYLCHOLINES, DIACYL**					
PC aa (32:2)	LC-MS/MS	4.3 ± 1.3	3.8 ± 1.1	1.13	0.18
PC aa (36:6)	LC-MS/MS	0.7 ± 0.2	0.6 ± 0.2	1.17	0.22
PC aa (36:0)	LC-MS/MS	6.05 ± 1.4	6.18 ± 1.4	0.98	−0.03
PC aa (38:6)	LC-MS/MS	1.007 ± 0.284	0.901 ± 0.194	1.12	0.16
PC aa (38:0)	LC-MS/MS	0.801 ± 0.162	0.831 ± 0.161	0.96	−0.05
PC aa (40:6)	LC-MS/MS	1.6 ± 0.4	1.7 ± 0.4	0.96	−0.05
PC aa (40:2)	LC-MS/MS	0.367 ± 0.061	0.376 ± 0.061	0.95	−0.08
PC aa (40:1)	LC-MS/MS	0.209 ± 0.034	0.214 ± 0.044	0.98	−0.03
**LYSOPHOSPHATIDYLCHOLINES, ACYL C**					
LysoPC(14:0)	LC-MS/MS	0.83 ± 0.12	0.77 ± 0.11	1.08	0.11
LysoPC(16:1)	LC-MS/MS	0.63 ± 0.14	0.63 ± 0.11	1.00	0.00
LysoPC(16:0)	LC-MS/MS	20 ± 4	19 ± 3	1.05	0.07
LysoPC(17:0)	LC-MS/MS	2.83 ± 0.61	2.86 ± 0.41	0.99	−0.02
LysoPC(18:2)	LC-MS/MS	16 ± 4	14 ± 2	1.14	0.19
LysoPC(18:1)	LC-MS/MS	6.5 ± 1.4	6.4 ± 1.1	1.02	0.02
LysoPC(18:0)	LC-MS/MS	29 ± 6	30 ± 3	0.97	−0.05
LysoPC(20:4)	LC-MS/MS	0.51 ± 0.14	0.44 ± 0.11	1.17	0.23
LysoPC(20:3)	LC-MS/MS	1.7 ± 0.4	1.6 ± 0.3	1.06	0.09
LysoPC(24:0)	LC-MS/MS	0.051 ± 0.014	0.051 ± 0.011	1.00	0.00
LysoPC(26:1)	LC-MS/MS	0.109 ± 0.051	0.095 ± 0.042	1.15	0.20
LysoPC(26:0)	LC-MS/MS	0.9 ± 0.3	0.6 ± 0.3	1.50	0.58
LysoPC(28:1)	LC-MS/MS	0.349 ± 0.122	0.266 ± 0.064	1.30	0.37
LysoPC(28:0) *	LC-MS/MS	0.322 ± 0.121	0.228 ± 0.044	1.41	0.50
**SPHINGOMYELINS**					
SM(16:1)	LC-MS/MS	6 ± 1	5 ± 1	1.10	0.13
SM(16:0)	LC-MS/MS	69 ± 10	65 ± 9	1.06	0.09
SM(18:1)	LC-MS/MS	11 ± 3	9 ± 2	1.22	0.29
SM(18:0)	LC-MS/MS	12 ± 1	11 ± 2	1.09	0.13
SM(20:2) *	LC-MS/MS	1.2 ± 0.3	0.9 ± 0.2	1.33	0.42
**HYDROXYSPHINGOMYELINS**					
SM(14:1(OH))	LC-MS/MS	5.6 ± 1.2	5.1 ± 1.1	1.10	0.13
SM(16:1(OH))	LC-MS/MS	9 ± 1	8 ± 2	1.13	0.17
SM(22:2(OH))	LC-MS/MS	5 ± 1	4 ± 1	1.10	0.13
SM(22:1(OH))	LC-MS/MS	9.3 ± 1.4	8.8 ± 1.4	1.06	0.08
SM(24:1(OH))	LC-MS/MS	1.9 ± 0.4	1.9 ± 0.4	1.00	0.00
**ACYLCARNITINES**					
C0 (Carnitine) *	LC-MS/MS	8 ± 2	7 ± 1	1.16	0.22
C2 (Acetylcarnitine)	LC-MS/MS	1.84 ± 0.81	1.54 ± 0.44	1.19	0.25
C3:1 (Propenoylcarnitine)	LC-MS/MS	0.028 ± 0.004	0.029 ± 0.004	0.97	−0.05
C3 (Propionylcarnitine) *	LC-MS/MS	0.22 ± 0.052	0.18 ± 0.014	1.22	0.29
C4:1 (Butenylcarnitine)	LC-MS/MS	0.017 ± 0.002	0.017 ± 0.002	1.00	0.00
C4 (Butyrylcarnitine) *	LC-MS/MS	0.197 ± 0.041	0.143 ± 0.034	1.38	0.46
C3-OH (Hydroxypropionylcarnitine)	LC-MS/MS	0.027 ± 0.004	0.028 ± 0.004	0.96	−0.05
C5:1 (Tiglylcarnitine)	LC-MS/MS	0.023 ± 0.004	0.023 ± 0.004	1.00	0.00
C5 (Valerylcarnitine)	LC-MS/MS	0.11 ± 0.034	0.08 ± 0.014	1.38	0.46
C4-OH (C3-DC) (Hydroxybutyrylcarnitine)	LC-MS/MS	0.041 ± 0.004	0.042 ± 0.004	0.98	−0.03
C6:1 (Hexenoylcarnitine)	LC-MS/MS	0.023 ± 0.004	0.023 ± 0.004	1.00	0.00
C6 (C4:1-DC) (Hexanoylcarnitine)	LC-MS/MS	0.053 ± 0.014	0.049 ± 0.011	1.08	0.11
C5-OH (C3-DC-M) (hydroxyvalerylcarnitine)	LC-MS/MS	0.038 ± 0.004	0.036 ± 0.004	1.06	0.08
C5:1-DC (Glutaconylcarnitine)	LC-MS/MS	0.018 ± 0.003	0.018 ± 0.003	1.00	0.00
C5-DC (C6-OH)(Glutarylcarnitine)	LC-MS/MS	0.028 ± 0.004	0.027 ± 0.004	1.04	0.05
C8 (Octanoylcarnitine)	LC-MS/MS	0.019 ± 0.011	0.018 ± 0.004	1.06	0.08
C5-M-DC (methylglutarylcarnitine)	LC-MS/MS	0.019 ± 0.002	0.019 ± 0.003	1.00	0.00
C9 (Nonaylcarnitine)	LC-MS/MS	0.022 ± 0.002	0.021 ± 0.003	1.05	0.07
C7-DC (Pimelylcarnitine)	LC-MS/MS	0.037 ± 0.042	0.026 ± 0.031	1.42	0.51
C10:2 (Decadienylcarnitine)	LC-MS/MS	0.05 ± 0.01	0.06 ± 0.01	0.89	−0.18
C10:1 (Decenoylcarnitine)	LC-MS/MS	0.172 ± 0.032	0.163 ± 0.034	1.06	0.08
C10 (Decanoylcarnitine)	LC-MS/MS	0.19 ± 0.04	0.18 ± 0.03	1.06	0.08
C12:1 (Dodecenoylcarnitine)	LC-MS/MS	0.085 ± 0.013	0.081 ± 0.014	1.05	0.07
C12 (Dodecanoylcarnitine)	LC-MS/MS	0.038 ± 0.011	0.035 ± 0.003	1.09	0.12
C14:2 (Tetradecadienylcarnitine)	LC-MS/MS	0.036 ± 0.004	0.033 ± 0.004	1.09	0.13
C14:1 (Tetradecenoylcarnitine)	LC-MS/MS	0.06 ± 0.01	0.05 ± 0.01	1.13	0.17
C14 (Tetradecanoylcarnitine)	LC-MS/MS	0.018 ± 0.011	0.015 ± 0.004	1.20	0.26
C12-DC (Dodecanedioylcarnitine)	LC-MS/MS	0.018 ± 0.002	0.019 ± 0.003	0.95	−0.08
C14:2-OH (Hydroxytetradecadienylcarnitine)	LC-MS/MS	0.0079 ± 0.0021	0.0075 ± 0.0011	1.05	0.07
C14:1-OH (Hydroxytetradecenoylcarnitine)	LC-MS/MS	0.008 ± 0.002	0.009 ± 0.001	0.89	−0.17
C16:2 (Hexadecadienylcarnitine)	LC-MS/MS	0.012 ± 0.002	0.012 ± 0.002	1.00	0.00
C16:1 (Hexadecenoylcarnitine)	LC-MS/MS	0.026 ± 0.004	0.025 ± 0.002	1.04	0.06
C16 (Hexadecanoylcarnitine)	LC-MS/MS	0.021 ± 0.011	0.019 ± 0.004	1.11	0.14
C16:2-OH (Hydroxyhexadecadienylcarnitine)	LC-MS/MS	0.005 ± 0.001	0.006 ± 0.001	0.83	−0.26
C16:1-OH (Hydroxyhexadecenoylcarnitine)	LC-MS/MS	0.018 ± 0.003	0.019 ± 0.004	0.95	−0.08
C16-OH (Hydroxyhexadecanoylcarnitine)	LC-MS/MS	0.007 ± 0.001	0.008 ± 0.001	0.88	−0.19
C18:2 (Octadecadienylcarnitine)	LC-MS/MS	0.006 ± 0.001	0.007 ± 0.001	0.86	−0.22
C18:1 (Octadecenoylcarnitine)	LC-MS/MS	0.014 ± 0.003	0.016 ± 0.003	0.88	−0.19
C18 (Octadecanoylcarnitine)	LC-MS/MS	0.022 ± 0.011	0.021 ± 0.004	1.10	0.14
C18:1-OH (Hydroxyoctadecenoylcarnitine)	LC-MS/MS	0.009 ± 0.001	0.008 ± 0.001	1.13	0.17
**METAL IONS**					
Sodium (Na)	ICP-MS	132919 ± 12091	134408 ± 16387	0.99	−0.02
Magnesium (Mg)	ICP-MS	920 ± 77	948 ± 104	0.97	−0.04
Phosphorus (P)	ICP-MS	1315 ± 193	1271 ± 111	1.03	0.05
Potassium (K)	ICP-MS	4283 ± 428	4315 ± 341	0.99	−0.01
Calcium (Ca)	ICP-MS	2251 ± 232	2193 ± 211	1.03	0.04
Iron (Fe)	ICP-MS	49 ± 14	57 ± 10	0.86	−0.22
Copper (Cu)	ICP-MS	8 ± 2	9 ± 2	0.89	−0.17
Zinc (Zn)	ICP-MS	13 ± 2	12 ± 1	1.05	0.07
Selenium (Se)	ICP-MS	1.4 ± 0.2	1.3 ± 0.2	1.08	0.11
Rubidium (Rb)	ICP-MS	1.8 ± 0.2	1.8 ± 0.2	1.00	0.00
Strontium (Sr)	ICP-MS	0.94 ± 0.14	0.98 ± 0.04	0.96	−0.06
Cesium (Cs) *	ICP-MS	0.0016 ± 0.0002	0.0019 ± 0.0003	0.84	−0.25
Barium (Ba)	ICP-MS	0.19 ± 0.04	0.21 ± 0.02	0.90	−0.14

^1^ Mean ± standard deviation * *p*-value < 0.05.

**Table 2:** Blood components associated with RFI as measured by different studies.

Metabolite	This Study	Fitzsimons et al., 2013 (Olympus Chemistry Analyzer)	Karisa et al., 2014_Discovery Population (NMR)	Karisa et al., 2014_Validation Population (NMR)	Jorge-Smeding et al., 2019 (LC-MS/MS)
Glucose *	L ^1^	H ^2^			
Urea	H	H			
Creatinine	L	L	L		
Creatine *	L		H (Glutamine overlap)	H (Glutamine overlap)	
Carnitine	H	H	H (Glutamine overlap)	H (Glutamine overlap)	
Formate	H	H	L		
Hydroxyisobutyrate	ND ^3^	H	H (Glucose overlap)	
Tyrosine	H	H	H		
Glycine	L	L	H		
Pantothenate	ND				
Hippurate	ND		H (Glutamine overlap)	L (Glutamine overlap)	
Threonine	H		H		
Acetate *	H		L		
Phenylalanine	H		H		
Lysine	H		H		
Citrate	ND		H		
Betaine	H		H		
Glutamate *	H		L		
Valine	H			H	H
Choline *	L			H	
Histidine	L			L	
Uridine	H			H	
2-methylamine	ND			L	
3-methylamine	ND			L	
2-hydroxybutyrate	ND			H	
3-hydroxybutyrate *	H			L	
4-hydroxybutyrate	ND			H (Acetone overlap)	
Succinate	ND			L (Mis-match)	
Oxo-butyrate	ND			L (Mis-match)	
Trans-4-hydroxy-L-proline	ND			L	
Proline	H			H	
Allantonin	ND			H (Mis-match)	
Glutamine	H = L			L (Overlap with glutamate, creatine, carnitine, hippurate)	
Aspartate	H				H
Ornitine *	L				H
Fumarate	H = L				L
Lysine	H				H

* Metabolites which concentration values do not agree with literature values; ^1^ L, LRFI; ^2^ H, HRFI; ^3^ ND, not detected.

**Table 3:** Nutrient analysis of barley-silage ration fed to bulls during the RFI test period in the GrowSafe system.

Diet Composition	Value
DM ^1^% (actual)	56.10
CP ^2^ (%DM)	14
ADF ^3^ (%DM)	25.25
NDF ^4^ (%DM)	40.50
TDN ^5^ (%DM)	69.60
Ca (%DM)	0.94
P (%DM)	0.34
Mg (%DM)	0.23
K (%DM)	1.38
Na (%DM)	0.13
Fe (PPM)	336
Mn (PPM)	70
Zn (PPM)	61
Cu (PPM)	16

^1^ DM, dry matter basis; ^2^ CP, crude protein; ^3^ ADF, acid detergent fibre; ^4^ NDF, neutral detergent fibre; ^5^ TDN, total digestible nutrients.
